# Da verdade factual à mentira organizada

**DOI:** 10.1590/0102-311XPT007524

**Published:** 2024-04-29

**Authors:** Neyson Pinheiro Freire, Isabel Cristina Kowal Olm Cunha

**Affiliations:** 1 Universidade Federal de São Paulo, São Paulo, Brasil.; 2 Conselho Federal de Enfermagem, Cuiabá, Brasil.

“*Contra fatos não há argumentos*”. O ditado popular, que por tanto tempo nos convenceu de que não há como contestar evidências ou provas concretas, já não tem a mesma contundência de antes. As *fake news* estão aí para colocá-lo em dúvida. Na era da pós-verdade, parece menos arriscado citar Nietzsche e sua famosa frase: “*não existem fatos, apenas interpretações*”.

É por essa linha de pensamento que o livro *Pós-verdade e Fake News: Como a Psicologia e a Comunicação são Usadas para Manipular o Mundo*^1^, de Márcio Sérgio Christino, leva-nos a um mergulho nas intrincadas ligações entre o fato, o boato e a mentira na trajetória da humanidade. Para confundir ainda mais quem inicia a leitura, o autor afirma, na apresentação da obra, que até mesmo a referência a Nietzsche é equivocada: “*Nietzsche não nega a existência de fatos, questiona apenas o modo pelo qual o conhecimento do fato é mediado*” (p. 11).

E os modos de mediação são muitos. Ao longo do livro, o autor vai conduzindo o leitor em uma jornada pela história da comunicação - da invenção da propaganda, passando pela chegada do telégrafo, o rádio e a TV, a internet, até a atualidade, com as redes sociais e a inteligência artificial. Dessa forma, ele vai desvendando como disseminar informações falsas têm sido uma estratégia recorrente empregada por líderes, governo e pessoas influentes em todos os tempos.

Na capa, retratos em cores quentes de Donald Trump, Vladimir Putin, Joseph Stalin e Adolf Hitler ilustram o fenômeno - e o autor realmente se aprofunda nesses quatro personagens. O livro se divide em três partes com títulos instigantes: *O Papa da Propaganda*, *Mais Americano do que o McDonald’s* e *A China vê Sherazade*. E, então, chega ao capítulo conclusivo: *O Mundo dos Mentirosos*. Na parte sobre propaganda, por exemplo, é oportuno relembrar como Joseph Goebbels, o homem da comunicação de Hitler, usou essa estratégia a favor do nazismo, a partir das teorias desenvolvidas por Edward Bernays, referência da propaganda no século XX. A inovação de Bernays foi associar palavras e ideias a emoções, buscando controlar os sentimentos do público e, com isso, manipular suas ações. Essas técnicas foram usadas para suscitar rejeição a determinadas ideias, paixões por outras, e até mesmo dependência psicológica a conceitos ou produtos.

Qualquer semelhança com os tempos atuais, portanto, não é coincidência. Em seu livro *Crystallizing Public Opinion* [Cristalizando a Opinião Pública] ^2^, Bernays apresenta conceitos que vigoram com mais força atualmente. Um deles: em vez de combater determinadas crenças, é mais eficaz desacreditar as autoridades que as estabeleceram. É o que temos visto acontecer no campo da saúde pública, em que a desconfiança gerada pela pós-verdade, em muitos casos, vem minando a credibilidade de profissionais e instituições de saúde.

Procurador de justiça do Ministério Público de São Paulo, o autor, Márcio Sérgio Christino, é uma das maiores autoridades brasileiras no combate ao crime organizado. Publicou também *Laços de Sangue: A História Secreta do PCC*^3^, em parceria com o jornalista Claudio Tognolli. Agora, em escrita solo, lança luz à guerra das narrativas, mostrando que “pós-verdade” e “fake news” não são apenas termos ou fenômenos contemporâneos, mas estão fortemente ligados à psique do ser humano.

Em uma análise minuciosa, a obra vai revelando como, em qualquer campo, os fatos podem ser distorcidos, modificados, “*sempre que houver um interesse humano e, sobretudo, pelo exercício do poder*” (p. 11).

Embora o foco do autor seja na política, revelando bastidores de governos e guerras, o livro leva a uma reflexão ampla sobre o tema. É inevitável não pensar nos efeitos devastadores das *fake news* na saúde pública. Um dos maiores desafios enfrentados nesse campo, na última década, é o surgimento de teorias da conspiração, como as que questionam a eficácia de vacinas. Durante a pandemia de COVID-19, a disseminação de *fake news* em grande escala levou a Organização Mundial da Saúde (OMS) a cunhar o termo “infodemia” ^4^, visto que o volume de desinformação comprometeu a adesão a medidas sanitárias, agravando a propagação do vírus e impactando negativamente a resposta da sociedade à crise ^5^.

Um dos pontos fortes do livro reside em questões cruciais, colocadas pelo autor logo no início da leitura, que também nos levam à saúde pública: “*o contraponto da verdade é a mentira, mas qual o contraponto do fato?*” (p. 12). Na abordagem do autor, a verdade foi sendo reduzida a outra natureza, a da “opinião”, que é um pensamento individual que se torna público a respeito de um fato, um dado de realidade. Daí o eterno conflito com a verdade factual, que detona um novo fenômeno, denominado mentira organizada, que é devastador diante da mentira tradicional e se tornou a “argamassa” da pós-verdade.

Diante da mentira organizada, afirma Christino, os indivíduos se tornam convertidos. E, nesse ponto, perde-se qualquer tipo de escrúpulo: vale fabricar imagens, testemunhas, deturpar falas, ou seja, criar *fake news*. Como o livro vai mostrando, com a ascensão de novos meios de comunicação e das redes sociais, a informação é diabolicamente moldada para articular o comportamento da sociedade e conduzir a narrativa dos fatos.

O resultado é um mundo em que as pessoas sabem cada vez menos o que é real e o que é invenção, a opinião individual reina sobre qualquer hipótese, o debate tem seu valor comprometido e os mentirosos ganham cada vez mais poder. Até porque “*a mentira é sempre mais atrativa, mais fácil de ser assimilada, criada ao gosto de quem busca convencer*” (p. 13).

A falta de discernimento entre o factual e ficcional compromete a autonomia das pessoas em relação à sua própria saúde, tornando-as suscetíveis a escolhas baseadas em mitos e concepções errôneas. Quando esse fenômeno se estende à saúde pública, contudo, coloca vidas em risco. Vide os resultados da pandemia, em que as *fake news* prejudicaram a adesão às medidas sanitárias. A verdade foi relativizada e a disputa de narrativas contaminou o debate público. Esse ambiente de (des)informação prejudicou as respostas sanitárias e impactou negativamente o trabalho dos profissionais de saúde da linha de frente ^6^.

*Pós-verdade e Fake News: Como a Psicologia e a Comunicação são Usadas para Manipular o Mundo*, portanto, é uma leitura esclarecedora, abordando conceitos complexos da psicologia, a fim de explicar como chegamos a esse nível de influência, como a manipulação da verdade moldou a história e segue influenciando a sociedade moderna. Até mesmo a liberdade, alardeada por grupos de direita e extrema direita, como uma maneira de justificar a expressão de conceitos duvidosos, é tratada de maneira contundente.

É importante destacar que a obra não apenas identifica problemas, mas também leva ao pensamento crítico. Christino oferece *insights* valiosos sobre como enfrentar os desafios da desinformação e preservar a integridade do debate público. “*O grande problema é que a conscientização depende primeiro da vontade do indivíduo e, depois, da disseminação do conhecimento necessário para uma avaliação*”, conclui o autor (p. 311).

A abordagem instiga a reflexão sobre os caminhos para mitigar os impactos prejudiciais da mentira organizada. Se, por um lado, já se sabe que é impossível eliminar as *fake news*, por outro, há a consciência de que é possível controlá-las em níveis menos prejudiciais à coletividade. Ao fim, cabe ao leitor expandir sua percepção do grau de exposição e assimilação do bombardeio diário de informações e buscar ter alguma clareza diante dos argumentos que tentam combater os fatos.
